# Advances in the treatment of chronic myeloid leukemia

**DOI:** 10.1186/1741-7015-9-99

**Published:** 2011-08-26

**Authors:** Anna M Eiring, Jamshid S Khorashad, Kimberly Morley, Michael W Deininger

**Affiliations:** 1 Division of Hematology and Hematologic Malignancies, University of Utah Huntsman Cancer Institute, Salt Lake City, Utah, USA

## Abstract

Although imatinib is firmly established as an effective therapy for newly diagnosed patients with chronic myeloid leukemia (CML), the field continues to advance on several fronts. In this minireview we cover recent results of second generation tyrosine kinase inhibitors in newly diagnosed patients, investigate the state of strategies to discontinue therapy and report on new small molecule inhibitors to tackle resistant disease, focusing on agents that target the T315I mutant of BCR-ABL. As a result of these advances, standard of care in frontline therapy has started to gravitate toward dasatinib and nilotinib, although more observation is needed to fully support this. Stopping therapy altogether remains a matter of clinical trials, and more must be learned about the mechanisms underlying the persistence of leukemic cells with treatment. However, there is good news for patients with the T315I mutation, as effective drugs such as ponatinib are on their way to regulatory approval. Despite these promising data, accelerated or blastic phase disease remains a challenge, possibly due to BCR-ABL-independent resistance.

## Background

Chronic myeloid leukemia (CML) is a myeloproliferative neoplasm caused by BCR-ABL, a chimeric gene generated as a result of a reciprocal translocation [t(9;22)(q34;q11), cytogenetically visible as the Philadelphia chromosome (Ph)] that places sequences from the ABL gene from chromosome 9 downstream of the BCR gene on chromosome 22. The fact that tyrosine kinase activity of BCR-ABL is *conditio sine qua non *for the protein's ability to transform cells led to the development of small molecule tyrosine kinase inhibitors (TKIs) [1]. It is a little more than ten years ago that the first TKI, imatinib, was approved for the treatment of chronic myeloid leukemia (CML) patients who had failed prior therapy with interferon-α (IFN). Two years later, the International Randomized Study of Interferon and STI571 (IRIS) study demonstrated the superiority of imatinib over IFN/cytarabine (the standard drug therapy at the time), in newly diagnosed chronic phase patients, and led to its approval for first-line therapy [2]. Prior to the development of imatinib, effective treatment for CML was limited to a minority of patients. IFN-based regimens prolonged survival compared to hydroxyurea, with induced durable responses in 10-30% of patients [3,4]. However, this benefit was largely limited to patients with low risk according to Sokal and came at the expense of significant toxicity. Allogeneic hematopoietic stem cell transplant in first chronic phase from a matched related donor produced five-year disease-free survival rates of approximately 50%. However, transplant-related mortality and morbidity were considerable and many patients were not eligible due to co-morbidities or lack of a suitable donor [5]. All this changed radically with the advent of imatinib. We now have the luxury of asking questions that would have seemed presumptuous just ten years ago, foremost whether we can safely discontinue imatinib in patients whose disease is consistently undetectable by RT-PCR. The logical extension of this question is whether patients who remain molecularly negative in the absence of therapy are cured of their disease, and generally how we should define cure in this context. Imatinib also changed how CML treatment is monitored. The IRIS trial established complete cytogenetic response (CCyR) and major molecular response (MMR), defined as a 3-log reduction of BCR-ABL transcripts compared to a standardized baseline, as key milestones associated with excellent long-term outcome, and provided a rationale for using these surrogate endpoints in subsequent clinical trials [6].

Despite this unprecedented success, some clouds have appeared in the sky of imatinib. Concerns first arose when it became apparent that a substantial fraction of the IRIS patients had left the study for a variety of reasons, a fact that was not immediately appreciated from Kaplan-Meyer or cumulative response graphics [7]. Thus at a follow-up of eight years, only 55% of patients treated with first-line imatinib in the IRIS study were still receiving imatinib, while the remainder had discontinued therapy, mostly due to unsatisfactory therapeutic effect or toxicity [8].

As a result of these concerns, the presentation of 'patient disposition' at a given time of follow-up is increasingly seen as mandatory complement to overall survival (OS) and event free survival (EFS) estimates. Moreover, it has become clear that the results of imatinib therapy are significantly less favorable in the community setting. A report from the Hammersmith Hospital defined imatinib failure more broadly than the IRIS study as discontinuation of drug for any reason, including toxicity. Additionally, the lack of a major cytogenetic response was considered as failure, in line with recommendations by the European Leukemia Network (ELN) [9]. Using these criteria, EFS at 5 years was calculated at only 63% [10]. Even more alarming are results from a population-based study of CML patients in Northwestern Britain that encompassed all patients diagnosed with CML in a geographically defined area over a 3.5 year period. At 24 months, only half of the patients were in CCyR and receiving imatinib [11]. While imatinib resistance can be caused by several mechanisms, including mutations in the kinase domain of BCR-ABL, it is likely that lack of adherence to medication is a major underlying reason for these sobering data, possibly by promoting the emergence of resistant clones through suboptimal, non-lethal target inhibition [12,13]. Perhaps it should not come as a surprise that chronic oral cancer therapy is subject to the same compliance limitations as other chronic drug therapies, and this will not be different with other oral agents [13]. Here we review three frontiers of CML therapy: improvements in first-line treatment, the therapeutic objective of disease eradication and novel agents to overcome drug resistance.

### The changing paradigm of frontline therapy for chronic phase CML

Dasatinib and nilotinib are highly potent BCR-ABL inhibitors that were initially approved for the treatment of patients who had failed prior therapy, including imatinib. Both are active against imatinib-resistant mutants of BCR-ABL and induce durable cytogenetic responses in approximately 50-60% of chronic phase patients, while responses in advanced phases tend to be transient. Both agents were recently compared with imatinib in the frontline chronic phase setting. The Dasatinib Versus Imatinib Study In Treatment-naïve CML (DASISION) study tested dasatinib 100 mg daily versus imatinib 400 mg daily, whereas the Evaluating Nilotinib Efficacy and Safety in Clinical Trials-Newly Diagnosed Patients (ENESTnd) study compared two doses of nilotinib (400 mg twice daily and 300 mg twice daily) with imatinib 400 mg daily [14,15]. Both studies found the experimental arms superior in the primary endpoint (DASISION: confirmed CCyR by 12 months; ENESTnd: MMR at 12 months), and results were confirmed on a recent update (Table 1). Patients treated with nilotinib had a significantly reduced risk of progression, while no such difference was observed in the DASISION study. Based on these results, both nilotinib and dasatinib were approved for frontline therapy of newly diagnosed patients in the US and in some European countries. A third phase 3 trial (BELA): Bosutinib Efficacy and safety in newly diagnosed chronic myeloid LeukemiA) tested bosutinib, a second generation TKI not currently approved, versus imatinib in newly diagnosed patients. Surprisingly, this study failed to demonstrate superiority of the bosutinib arm in the primary endpoint, the rate of CCyR at 12 months. It seems therefore unlikely that the drug will be approved for frontline therapy [16]. There is suspicion that the disappointing results may be due to frequent dose interruptions for diarrhea, a common side effect of bosutinib, which might have been manageable with more aggressive supportive care. As many patients were treated in smaller centers, this is a warning that 'outsourcing' of clinical studies to less experienced centers can be problematic.

**Table 1:** Response rates in phase 3 studies comparing imatinib with nilotinib or dasatinib

	DASISION		ENESTnd		
Median follow-up (months)	18		NR		

Minimum follow-up (months)	16		24		

	Dasatinib 100 mg QD	Imatinib 400 mg QD	Nilotinib 300 mg BID	Nilotinib 400 mg BID	Imatinib 400 mg QD

	(N = 259)	(N = 260)	(N = 282)	(N = 281)	(N = 283)

Confirmed complete cytogenetic response by 18 mo (%)	78	70	NR	NR	NR

Major molecular response at any time (%)	57	41	71	67	44

Complete molecular response at any time (%)	13	7	26	21	10

Overall survival (%)	96.0	97.9	97.4	97.8	96.4

Progression-free survival (%)	94.9	93.7	98	97.7	95.2

Discontinued treatment (%)	19	20	26	22	33

Data are based on the update reported at the 2010 Annual Meeting of the American Society of Hematology in Orlando, FL. NR = not reported

Should all newly diagnosed patients be treated with a second generation inhibitor? Given the association between CCyR on imatinib and EFS and OS, it is hard to refute the logic of minimizing progression risk by reducing leukemia burden faster and more profoundly. One important factor is that the tolerability of the newer agents is at least comparable to that of imatinib. However, differences in OS have yet to be observed, albeit with limited follow-up. Another concern in both studies is that approximately 20% of patients had dropped out from the experimental arms for a variety of reasons. Additionally, EFS on imatinib is excellent in patients with low risk according to Sokal or Hasford score, suggesting that these patients may be safely managed with the less expensive drug, an issue that will become even more important once generic imatinib becomes available (likely in 2015). One would predict that the clinical importance of accurate molecular prognostication tools, such as gene expression profiling, will increase proportionately to the price difference between alternative therapeutic options [17].

Which parameters will guide the selection of dasatinib or nilotinib in newly diagnosed patients? In the absence of a direct comparison between the two agents, and in view of their overall comparable efficacy, the selection of therapy is directed primarily toward minimizing the side effects. Both agents are generally well tolerated; however, conditions such as a history of GI bleeding or congestive heart failure favor nilotinib, which is relevant since the median age at diagnosis is 60 years. On the other hand, convenience may favor dasatinib due to the once daily dosing schedule and independence from meals, important aspects for patients with an irregular life style. Whether the different dosing regimens indeed translate into differences in adherence has not yet been studied.

### Eradicating the CML clone?

The most convincing argument for a switch to second generation TKIs would be the ability to eventually discontinue therapy in a larger fraction of patients. The French Stop Imatinib (STIM) study enrolled 100 CML patients who had been in complete molecular response (CMR: consistently negative BCR-ABL PCR using an assay with a sensitivity of 1:10^5^) for a minimum of two years prior to discontinuation of imatinib [18]. With a median follow-up of 17 months, 54 patients had experienced a recurrence, with the majority relapsing during the first six months. The overall probability of maintaining a CMR at 12 months was 43%, and in the sixty-nine patients followed for more than 12 months, the recurrence-free survival was 41% and 38% at one and two years, respectively. Female sex, higher Sokal risk score, and shorter duration of therapy were all associated with recurrence, while previous treatment with IFN did not affect relapse rates. Similar results were reported in a smaller Australian study [19]. One can only speculate about the eventual outcome of these trials. All patients may eventually experience a recurrence, or there may be a subset of patients who maintain CMR long-term. Given that the sensitivity of any assay to detect residual leukemia is eventually limited, we will never know whether such patients are 'cured', implying that an operational definition of cure is required, perhaps as a risk of developing clinical CML that is not different from the risk of the general population. The hope is now that second generation TKIs will allow for permanent discontinuation of therapy in a larger proportion of patients. Indeed, the DASISION and ENESTnd studies showed higher rates of CMR in the experimental arms (Table 1). On the other hand, one could argue that the overall rate of CMR is lower than would be expected from the very rapid decline of leukemia burden, suggesting that in most patients the residual population of CML cells is beyond the reach of TKIs, consistent with the observation that primitive CML cells maintain viability despite TKI-induced inhibition of BCR-ABL [20].

If CML stem cells are innately resistant to TKIs, can they be targeted with drug combinations? The most promising results have been reported from the SPIRIT study, which tested 400 mg and 600 mg imatinib daily vs. combinations of 400 mg imatinib with pegylated IFN-α-2a or cytarabine. At 12-months, the rates of MMR and CMR were significantly higher in the imatinib/pegylated IFN-α-2a arm compared to all other arms [21]. Similar results were seen in the Nordic CML study, which used a comparable combination, but not in the German CML IV trial, which used conventional IFN in combination with imatinib [22,23]. It is tempting to speculate that the type of IFN is responsible for the discrepant results, highlighting the fact that every detail matters. On the other hand, given that no difference in EFS or OS has been observed thus far in any of the studies, the 'real world' impact of these findings remains to be seen with longer follow-up. Other agents currently in early clinical testing in combination with TKIs include inhibitors of the Hedgehog pathway, inhibitors of authophagy, histone deacetylase inhibitors and others.

### New options for patients with drug resistance

Dasatinib and nilotinib are active in patients with imatinib failure. As with any other therapy for CML, responses are generally durable in chronic phase, but only transient in accelerated or blastic phase. While point mutations in the BCR-ABL kinase domain are the best characterized mechanism of resistance, it has become increasingly clear that resistance is more complex. This is supported by at least two lines of evidence. Firstly, many patients with resistance, particularly primary resistance in chronic phase, do not have BCR-ABL kinase domain mutations [24]. Secondly, with the exception of the pan-resistant T315I mutant, there is only weak correlation between in vitro sensitivity and in vivo response, indicating that additional mechanisms must in part govern responses, including mechanisms that are BCR-ABL-independent [25,26]. It is likely that the true prevalence of BCR-ABL-independent resistance will be known only when a TKI with activity against all mutants of BCR-ABL, including T315I, is available and widely used. Two agents have emerged that might test this hypothesis. Ponatinib (formerly AP24534) is a multitargeted kinase inhibitor that is active against all BCR-ABL mutants tested, including T315I. In vitro mutagenesis screens failed to reveal any new single mutation liability, in contrast to second-line TKIs tested with the same experimental system [27]. In a phase I study that included mostly patients with Ph-positive leukemia who had failed at least two TKIs, more than 50% of patients in chronic phase attained CCyR. Remarkably, the rate was close to 100% in patients with the T315I mutation, transforming a prognostically unfavorable biomarker into a predictor of favorable response [28]. As always, responses in patients with advanced disease were less frequent, less profound and less stable. Although the mechanisms underlying ponatinib resistance have not been studied, it is possible that BCR-ABL-independent resistance will become common. Alternatively, as yet unidentified composite mutations may play a role, either alone or in combination with conventional mechanisms, such as drug efflux and BCR-ABL amplification. A phase II study of ponatinib is currently ongoing and may shed first light on this issue. Another mechanistically different BCR-ABL kinase inhibitor is DCC-2036. This compound binds to the switch pocket, an allosteric site that controls the conformational changes that are required for the kinase to 'breath', allowing for repeated cycles of ATP and substrate interaction. Like Ponatinib, DCC-2036 is active against a broad spectrum of kinase domain mutants, including T315I, and mutagenesis assays show near-complete suppression of resistant clone outgrowth at high drug concentrations [29]. A phase I study is currently recruiting, but results have not yet been presented.

## Conclusion

The landscape of CML management has changed considerably since approval of imatinib. Long-term survival is a reality for the majority of patients, and one could argue that there would be much less demand for new therapies if patients were more compliant or physicians were better at managing side effects. In 2011 we have the privilege of witnessing improvements to first-line therapy using second generation TKIs, while third-line TKIs emerge as an effective salvage for patients who fail nilotinib and dasatinib, including those with the T315I mutation. It is easy to predict that the next quantum leap will be the ability to discontinue therapy altogether. For now, this option is limited to few selected patients, but the hope is that this population will grow with frontline use of dasatinib or nilotinib. However, some skepticism seems in order and it is conceivable that for the majority of patients, disease eradiation is beyond the reach of TKIs. Time will tell whether combinations with other signal transduction inhibitors or old-fashioned IFN might achieve this end result.

## Competing interests

The authors declare that they have no competing interests.

## Authors' contributions

AME, JSK and MWD jointly wrote the manuscript. KM provided additional support.

## Pre-publication history

The pre-publication history for this paper can be accessed here:

http://www.biomedcentral.com/1741-7015/9/99/prepub
